# Ferroptosis‐related gene signature as a prognostic marker for lower‐grade gliomas

**DOI:** 10.1111/jcmm.16368

**Published:** 2021-02-16

**Authors:** Yi Zheng, Qiang Ji, Lei Xie, Can Wang, Chun‐Na Yu, Ya‐Li Wang, Jing Jiang, Feng Chen, Wen‐Bin Li

**Affiliations:** ^1^ Department of Neuro‐Oncology Cancer Center Beijing Tiantan Hospital Capital Medical University Beijing China; ^2^ Department of Neurosurgery The Third Affiliated Hospital of Hebei Medical University Shijiazhuang China

**Keywords:** ferroptosis, lower‐grade gliomas, prognosis

## Abstract

Ferroptosis is a newly discovered form of programmed cell death, which has unique biological effects on metabolism and redox biology. In this study, the prognostic value of ferroptosis‐related genes was investigated in lower‐grade gliomas (LGG). We downloaded the ferroptosis‐related genes from the FerrDb dataset. Univariate Cox and LASSO regression analyses were applied to identify genes correlated with overall survival (OS). Subsequently, 12 ferroptosis‐related genes were screened to establish the prognostic signature using stepwise multivariate Cox regression. According to the median value of risk scores, patients were divided into low‐ and high‐risk subgroups. The Kaplan‐Meier curves showed the high‐risk group had a lower OS. The predictive power of the risk model was validated using the CGGA. Functional analysis revealed that the terms associated with plasma membrane receptor complex, immune response and glutamate metabolic process were primarily related to the risk model. Moreover, we established a nomogram that had a strong forecasting ability for the 1‐, 3‐ and 5‐year OS. In addition, we compared the risk scores between different clinical features. We also detected infiltration of macrophages and monocytes in different subgroups. Overall, our study identified the prognostic signature of 12 ferroptosis‐related genes, which has the potential to predict the prognosis of LGG.

## 1 INTRODUCTION

Lower‐grade gliomas (LGG) are an important class of primary malignant tumours in the central nervous system, accounting for about 20% of all primary brain tumours.^1^ The standard treatment regimen primarily involves surgical resection, with a post‐operative recurrence risk assessment, and high‐risk patients receive adjuvant chemoradiation.^2^ However, the clinical outcomes of LGG are highly variable. Emerging evidence shows that molecular signature alterations correlate more significantly with prognosis and predictive markers than do histopathological alterations.^3^, ^4^ Isocitrate dehydrogenase 1 (IDH1) mutation, 1p/19q codeletion and O6‐methylguanine‐DNA methyltransferase (MGMT) promoter methylation are widely utilized for molecular analysis in gliomas, but are insufficient for the precise prediction of prognosis.^5^, ^6^ Thus, novel biomarkers for risk stratification need to be identified.

Ferroptosis, characterized by the accumulation of lipid reactive oxygen species (lipid‐ROS), is an iron‐dependent form of cell death associated with the imbalance of redox homeostasis.^7^ The cystine/glutamate antiporter (system Xc‐) is a redox state regulator on the surface of the cellular plasma membrane,^8^ which can regulate the exchange of intracellular glutamate and extracellular cystine.^9^ Cystine promotes the synthesis of glutathione (GSH), which plays an important role as an intracellular antioxidant.^10^ Certain molecules deplete GSH by inhibiting the function of system Xc‐, leading to the accumulation of iron‐dependent lipid‐ROS.^11^ Tumour cells, enriched in free iron and with high levels of ROS, are more sensitive to ferroptosis. Recent studies have reported the association of ferroptosis with glioma cell proliferation, invasion and angiogenesis.^12^, ^13^, ^14^ However, there are few studies on its correlation with the prognostic value in patients with LGG.

**FIGURE 1:**
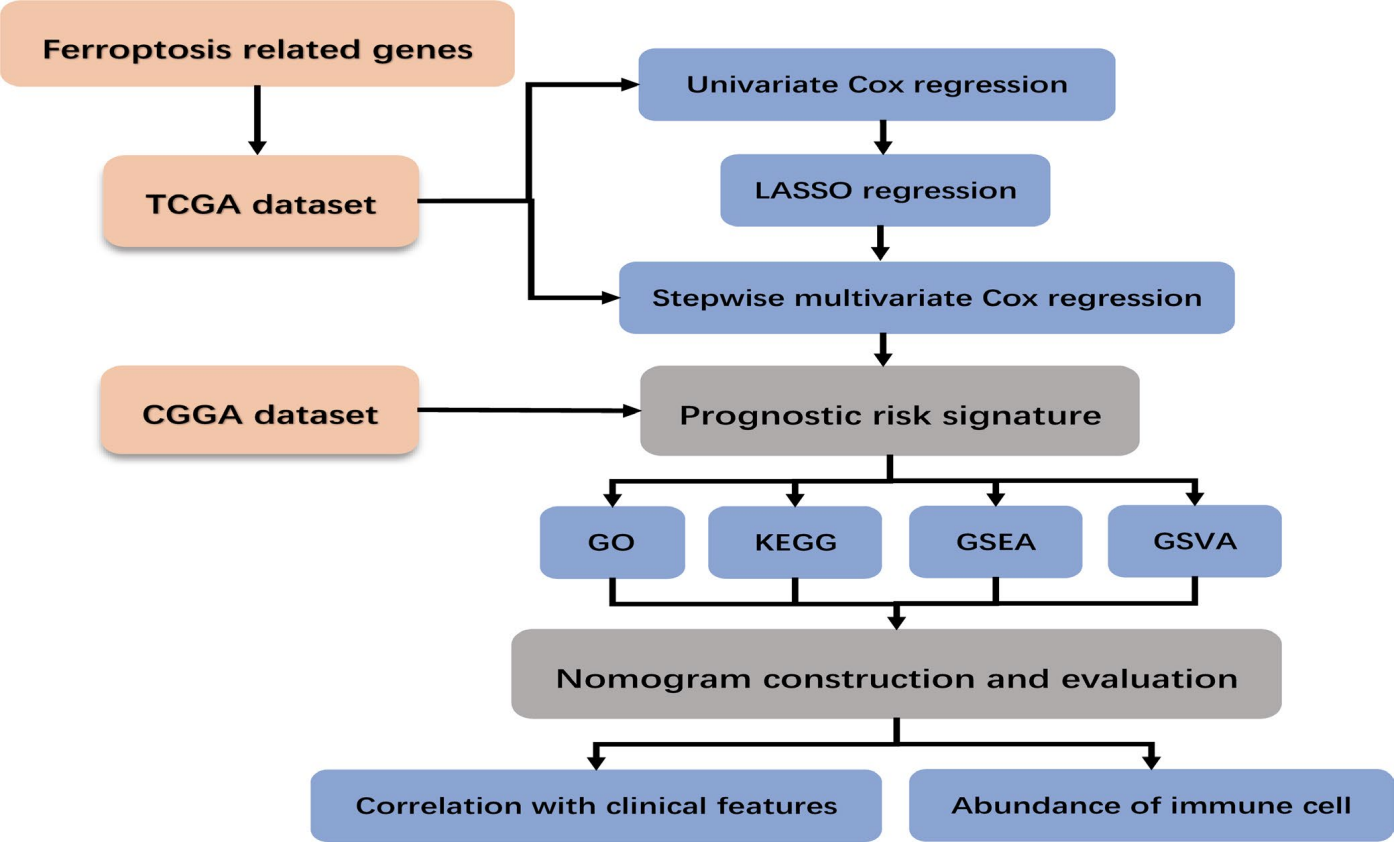
Flowchart of the construction of the ferroptosis‐related genes prognostic signature

In this study, we collected the RNA‐Seq data and clinical information of LGG in The Cancer Genome Atlas (TCGA). We identified 12 ferroptosis‐related genes by statistical analysis to establish a prognostic risk model. Meanwhile, patients with LGG in the Chinese Glioma Genome Atlas (CGGA) database were selected as a validation cohort. Gene Ontology (GO),^15^, ^16^ Kyoto Encyclopedia of Genes and Genomes (KEGG),^17^ Gene Set Enrichment Analysis (GSEA) and Gene Set Variation Analysis (GSVA) were used to screen the functions and pathways enriched between high‐risk and low‐risk subgroups. Furthermore, we developed a nomogram model based on risk scores and clinical features to assess prognosis. Our results demonstrated that the ferroptosis‐related risk model was a potential prognostic marker and therapeutic target for LGG. The overview workflow is presented in Figure 1.

## 2 MATERIALS AND METHODS

### 2.1 Download of datasets and collection of ferroptosis‐related genes

The gene expression RNA‐Seq (HTSeq‐FPKM) and clinical information of 495 patients with LGG in TCGA were collected from the University of California Santa Cruz (UCSC) Xena (https://xenabrowser.net/datapages/). Furthermore, 590 LGG samples were downloaded from the CGGA (http://cgga.org.cn/). In addition, the exclusion criteria included patients with a follow‐up of less than 30 days and a single gene with a total expression value of less than 10 in all 495 samples. We downloaded 259 ferroptosis‐related genes from the FerrDb data set (http://www.zhounan.org/ferrdb/).

### 2.2 Construction and validation of the prognostic risk model

A univariate Cox regression analysis was used to screen genes associated with prognosis, and we considered *P* < .01 as statistically significant. The least absolute shrinkage and selection operator (LASSO) regression was used to reduce the potential risk for overfitting with the ‘glmnet’ package in R.^18^ Ten‐fold cross‐validation (10FCV) was performed to select the optimal value of λ, and 22 key genes were obtained. We then used the stepwise multivariate Cox regression to perform the prognostic signature with 12 prognostic genes and drew a forest map to visualize the result.

**FIGURE 2:**
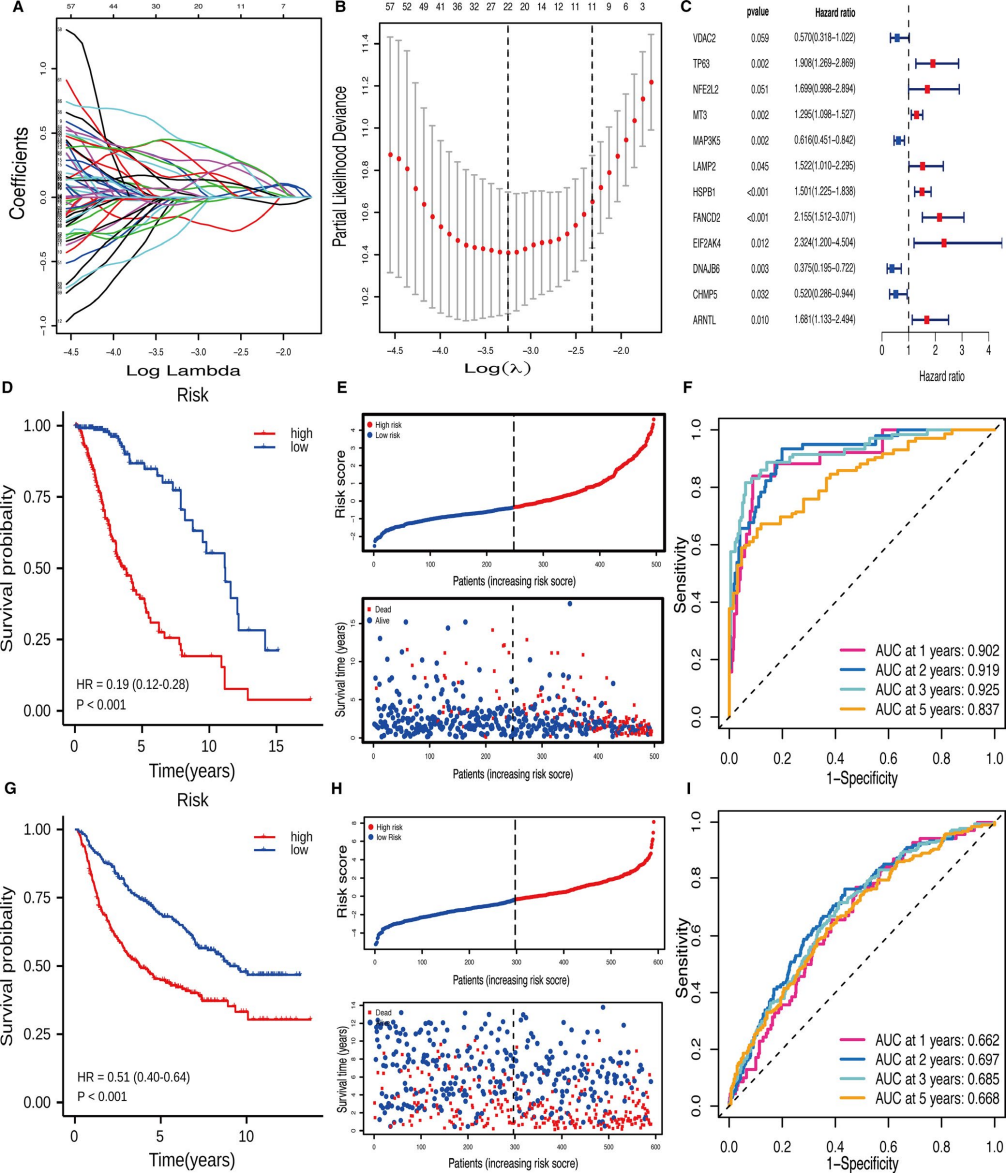
The construction and validation of the prognostic signature. (A‐B) LASSO regression was performed to select the optimal value of λ. (C) Stepwise multivariate Cox regression was applied to establish the prognostic signature. (D) Kaplan‐Meier survival curves showed that the low‐risk group had better OS than high‐risk group in TCGA. (E) The risk and survival status plots in TCGA. (F) ROC curves of the risk model for predicting the 1‐, 2‐, 3‐ and 5‐year OS in TCGA. (G) Kaplan‐Meier survival curves showed that the low‐risk group had better OS than high‐risk group in CGGA. (H) The risk and survival status plots in CGGA. (I) ROC curves of the risk model for predicting the 1‐, 2‐, 3‐ and 5‐year OS in CGGA

The formula

$$\text{Model}:\text{Riskscore}=\sum_{i=1}^{n}\beta_{i}*X_{i}$$

where n represents the total number of genes, *β*_i_ is the coefficient, and *X*_i_ is the expression value of the gene, was used to calculate the risk score: risk score = (*β*_1_*expression of gene *X*_1_) + (*β*_2_*expression of gene *X*_2_) + … (*β*_12_*expression of gene *X*_12_). Patients were clustered into low risk and high risk based on the median value of risk score in TCGA. Next, CGGA was used to verify the predictive power of the risk model. The Kaplan‐Meier survival curves and log‐rank tests were used to compare the differences in overall survival (OS) between the two categories. The risk and survival status plots are shown by the rank of the corresponding risk scores (Figure 2E,H). Finally, the 1‐, 2‐, 3‐ and 5‐year receiver operating characteristic (ROC) curves were plotted for TCGA and CGGA datasets to evaluate the sensitivity and specificity of survival prediction using ‘timeROC’ in R.

### 2.3 Identification and functional enrichment of differentially expressed genes

The expression of 12 signature genes and their corresponding clinical information are shown in the heat maps. We identified the differentially expressed genes (DEGs) using the Wilcoxon test between low‐ and high‐risk groups with FDR < 0.05 and |logFC| > 1. DEGs were analysed using the ‘clusterProfiler’ package in R for GO and KEGG. GSEA^19^ was conducted to confirm the activation or inhibition of biological processes and signalling pathways in low‐ and high‐risk groups. The KEGG gene set (C2.cp.kegg.v7.2.symbols.gmt) and GO gene set (C5.go.v7.2.symbols.gmt) were adopted from the Molecular Signatures Database (MSigDB). An FDR *q* < 0.25 and adjusted *P* < .05 were considered statistically significant. As a non‐parametric unsupervised analysis method, GSVA^20^ was used to assess the gene set enrichment (GSE) for each sample. We applied the ‘GSVA’ package in R to investigate significant metabolic and immunologic pathway differences between groups in the TCGA and CGGA datasets.

### 2.4 Nomogram construction and evaluation

Univariate and multivariate Cox analyses were used to screen for prognostic factors such as grade, radiation therapy, age, gender, IDH1 status, MGMT promoter status, 1p/19q codeletion and risk score. We filtered variables by stepwise regression to avoid the effect of multicollinearity.^21^ Subsequently, multivariate Cox regression analysis was used to identify the independent prognostic factors of the model. Thereafter, a nomogram was constructed, using the result of multi‐Cox analysis, to predict the 1‐, 3‐ and 5‐year OS in patients with LGG. This was then assessed by calibration curves. In addition, we compared the risk scores between different clinical features.

### 2.5 Estimation of the abundance of macrophages and monocytes by ImmuCellAI

ImmuCellAI (http://bioinfo.life.hust.edu.cn/ImmuCellAI#!/) can estimate the abundance of immune cell infiltration with higher accuracy than other algorithms.^22^ Using the ImmuCellAI website, we contrasted the infiltration levels of macrophages and monocytes between low‐ and high‐risk subgroups in TCGA and CGGA. *P* < .05 were accepted as statistically significant.

## 3 RESULTS

### 3.1 Construction of the ferroptosis‐related prognostic risk model

We searched for genes associated with ferroptosis in the FerrDb dataset. A total of 241 ferroptosis‐related genes were extracted in the TCGA‐LGG cohort. Univariate Cox and LASSO regression analyses were applied to screen for 22 feature genes (Figure 2A,B). Then, stepwise multivariate Cox regression analysis was used to select the best characteristic gene set and construct a regression model. Finally, the prognostic signature was established based on 12 ferroptosis‐related genes (Figure 2C). Their functions and coefficients are shown in Table S1. The cut‐off value of risk scores (−0.344) was used to dichotomize patients into low‐risk (n = 248) and high‐risk (n = 247) groups in the TCGA cohort. The prognosis was significantly better in the low‐risk group than in the high‐risk group (*P* <.001, HR = 0.19, 95% CI 0.12‐0.28; Figure 2D). As shown in Figure 2E, the distributions of risk score and survival status indicated that low‐risk scores were advantageous for survival. The ROC curves confirmed that the model had a good accuracy for predicting OS in TCGA (1‐year AUC = 0.902, 2‐year AUC = 0.919, 3‐year AUC = 0.925, 5‐year AUC = 0.837; Figure 2F).

### 3.2 Validation of the prognostic signature in the CGGA cohort

We examined the predictive power of the prognostic signature in the CGGA data set. It was divided into low‐risk (n = 297) and high‐risk (n = 293) groups using the same formula and cut‐off value as those for TCGA. As shown in Figure 2G, the low‐risk group had longer OS than the high‐risk group (*P* <.001, HR = 0.51, 95% CI 0.40‐0.64). The risk score and survival status distributions were similar to those in TCGA‐LGG (Figure 2H). The AUC values for 1‐, 2‐, 3‐ and 5‐year survival were 0.662, 0.697, 0.685 and 0.668 respectively (Figure 2I).

### 3.3 Functional analysis of risk model in TCGA and CGGA

**FIGURE 3:**
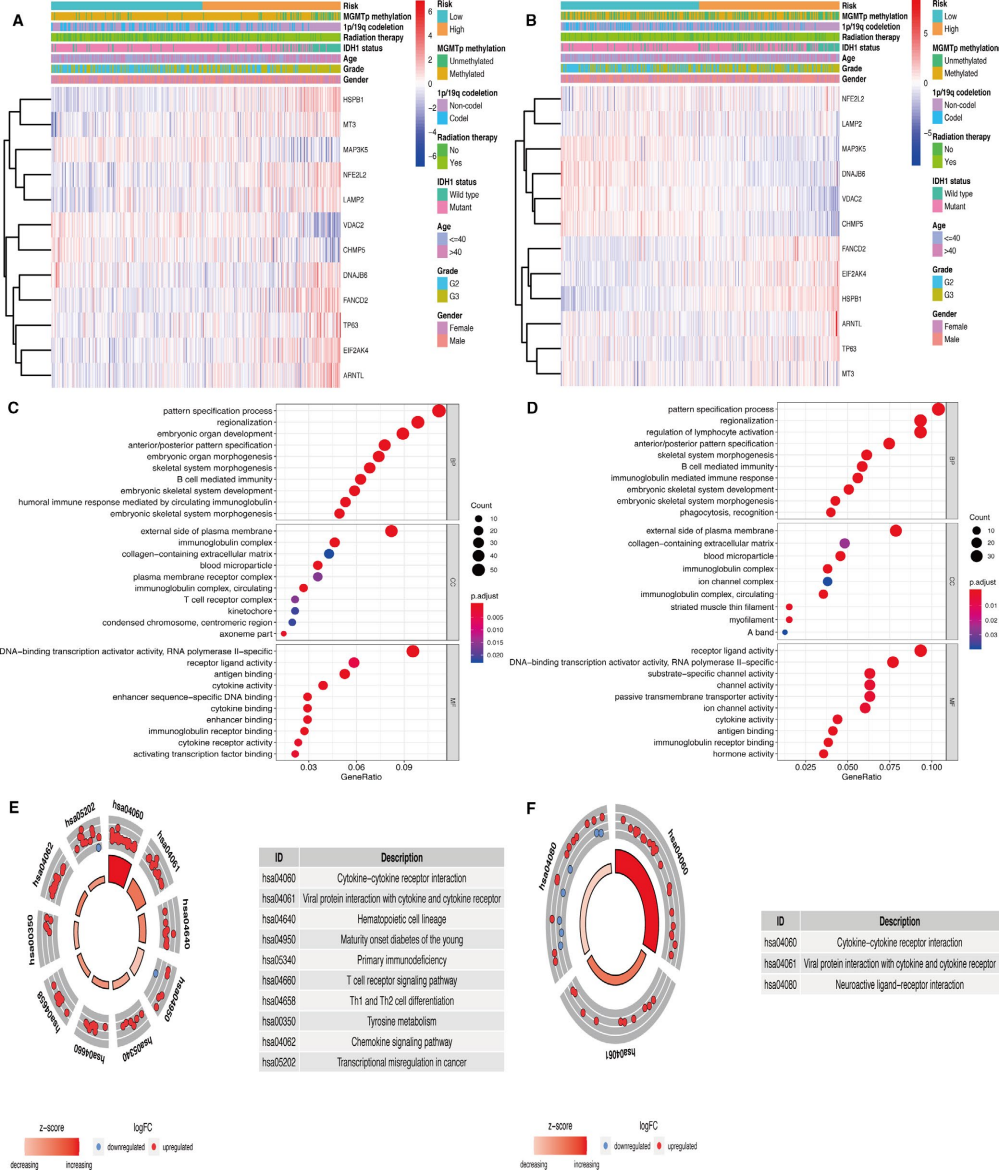
Heat maps and functional analysis related to the prognostic signature. (A‐B) Heat maps showed the 12 ferroptosis‐related genes and clinical information of patients in TCGA and CGGA. (C‐D) Go analysis based on DEGs in TCGA and CGGA. (E‐F) KEGG pathway analysis based on DEGs in TCGA and CGGA

The heat map of 12 genes in the ferroptosis‐related prognostic risk model, combined with clinical information, illustrated that patients in the high‐risk group had a tendency towards higher expression levels of risk genes and lower expression levels of protective genes (Figure 3A). These results were similar to those for CGGA (Figure 3B). A total of 1111 DEGs were identified between the high‐ and low‐risk groups in the TCGA cohort. GO analysis was primarily associated with the plasma membrane receptor complex, cytokine and immune response‐related terms (Figure 3C). KEGG analysis revealed that cytokine‐cytokine receptor interaction, T cell receptor signalling pathway, Th1 and Th2 cell differentiation, and tyrosine metabolism were obtained (Figure 3E). Furthermore, 806 DEGs were selected for GO and KEGG in the CGGA cohort. The results revealed that immune response, cytokine activity, receptor ligand activity and neuroactive ligand‐receptor interaction were primarily enriched (Figure 3D,F).

**FIGURE 4:**
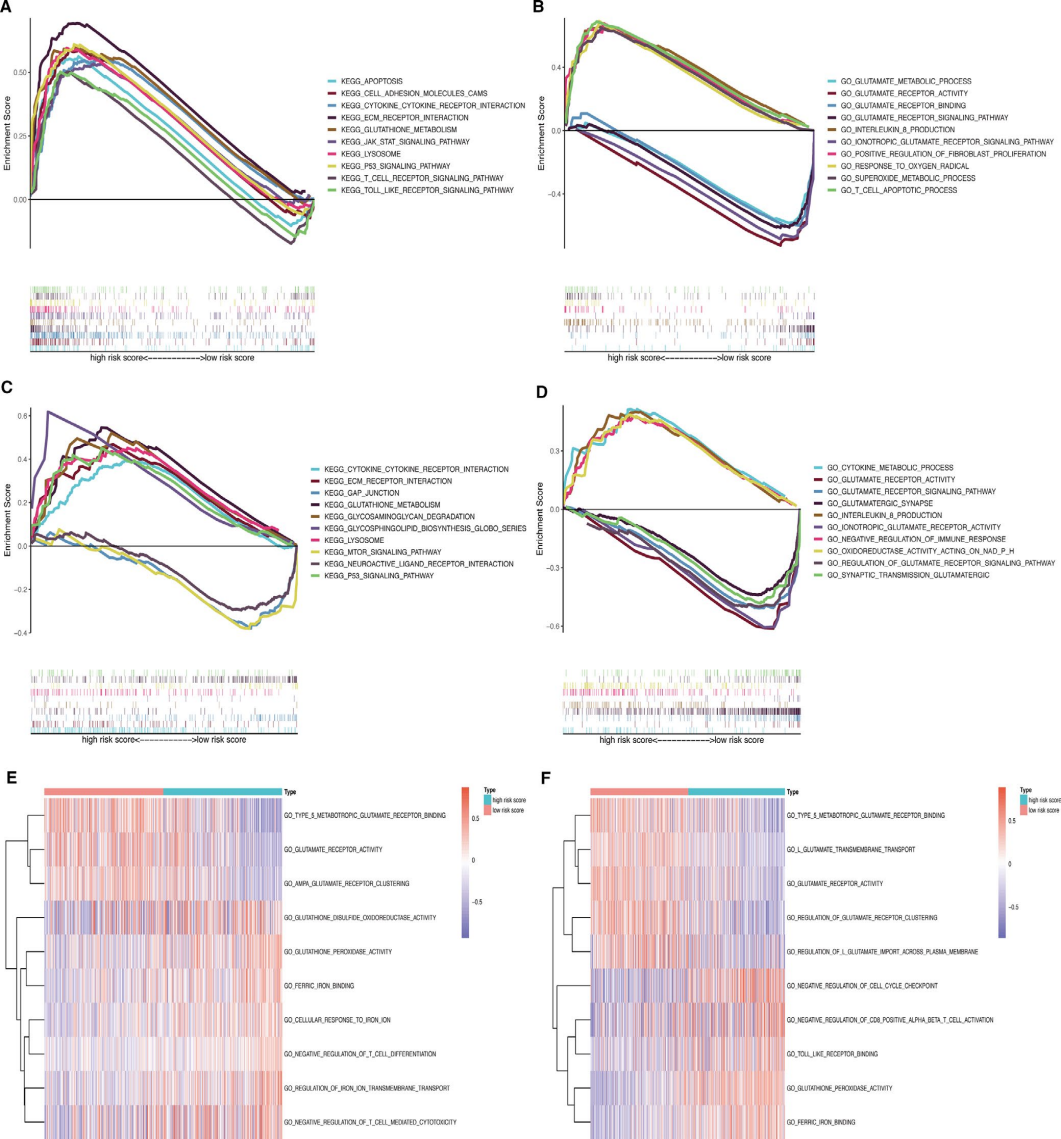
GSEA and GSVA for functional enrichment. (A–B) GSEA between low‐ and high‐risk groups in TCGA. (C–D) GSEA between low‐ and high‐risk groups in CGGA. (E–F) GSVA to assess the differences in pathways between different groups in TCGA and CGGA

GSEA analysis showed that the high‐risk group was associated with the pathways of glutathione metabolism and immune‐related functions from the KEGG database (Figure 4A). Among GO terms, the glutamate metabolic process, glutamate receptor binding, glutamate receptor activity, glutamate receptor signalling pathway and ionotropic glutamate receptor signalling pathway, which play vital roles in ferroptosis, were found to be enriched in the low‐risk group (Figure 4B). GSVA was used to identify the causes of the phenotypic differences. We found that the glutathione disulfide oxidoreductase activity, glutathione peroxidase activity, ferric iron binding, cellular response to iron ion, negative regulation of T cell differentiation, regulation of iron ion transmembrane transport and negative regulation of T cell–mediated cytotoxicity were enriched in the high‐risk group. Furthermore, type 5 metabotropic glutamate receptor binding, glutamate receptor activity and AMPA glutamate receptor clustering were more enriched in the low‐risk group (Figure 4E). These results were validated in CGGA (Figure 4C,D,F).

### 3.4 Development and validation of a nomogram

**FIGURE 5:**
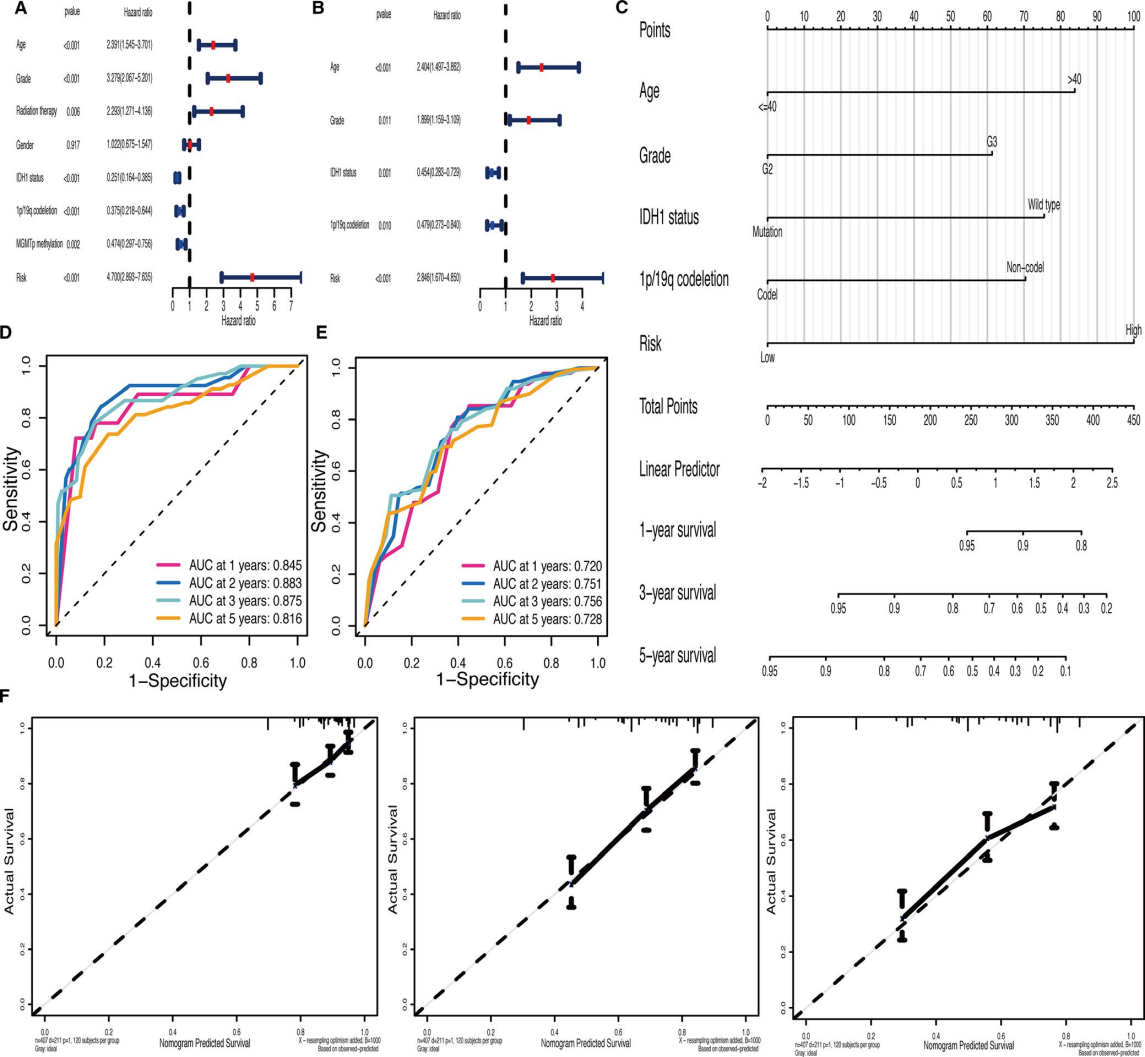
Development and validation of a nomogram. (A–B) Univariate and multivariate Cox regression analyses revealed that the risk score was an independent prognostic biomarker in TCGA. (C) Construction of the nomogram based on prognostic markers (age, grade, IDH1 status, 1p/19q codeletion status and risk). (D–E) The time‐dependent ROC curves of multivariate Cox model in TCGA and CGGA. (F) Calibration plot assessed the predicted power of OS at 1‐, 3‐ and 5‐ years in CGGA

Univariate and multivariate Cox regression analyses were performed to identify the risk score as an independent prognostic biomarker in LGG (Figure 5A,B). The nomogram used five prognostic markers (age, grade, IDH1 status, 1p/19q codeletion, and risk) to predict the 1‐, 3‐ and 5‐year OS in the TCGA cohort (Figure 5C). The patient was given a point based on the proportion of prognostic factors contributing to survival. Time‐dependent ROC curves were plotted to evaluate the prognostic capacity of the multivariate Cox model in TCGA (1‐year AUC = 0.845, 2‐year AUC = 0.883, 3‐year AUC = 0.875, 5‐year AUC = 0.816; Figure 5D) and CGGA (1‐year AUC = 0.720, 2‐year AUC = 0.751, 3‐year AUC = 0.756, 5‐year AUC = 0.728; Figure 5E). The calibration plot of validation set showed that the predicted power was close to the ideal curve (Figure 5F). In addition, the C‐index of the nomogram was 0.817 (95% CI 0.762‐0.872). Overall, these results demonstrated that the developed nomogram preforms well in predicting OS.

### 3.5 Correlation between clinical features and risk score

**FIGURE 6:**
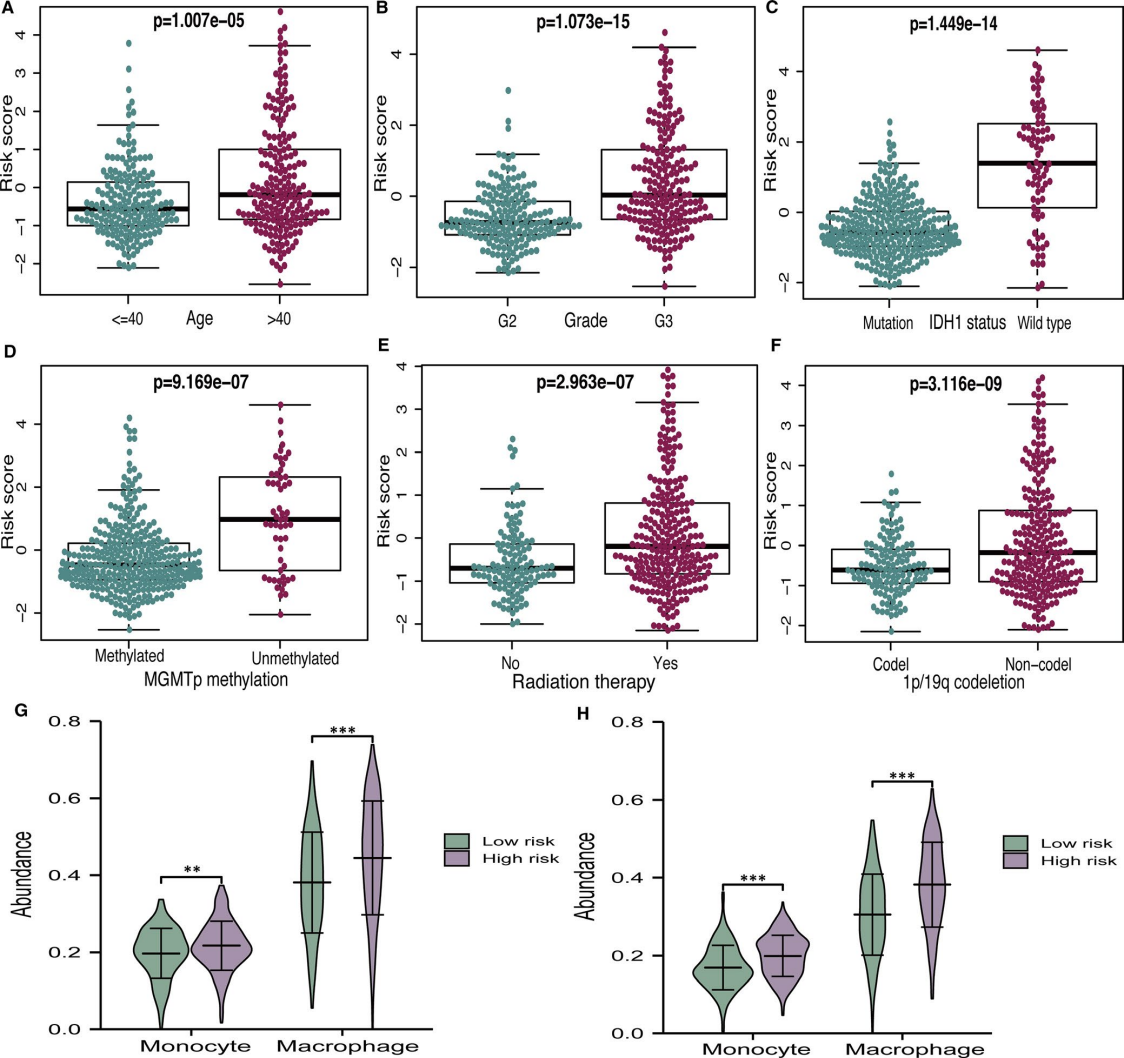
Relationship of risk score with different clinical features and immune cell infiltration. (A‐F) The correlations between risk score and different clinical features. (G–H) The differences between macrophage and monocyte infiltration in TCGA and CGGA

There were significant correlations between clinical parameters (age, grade, IDH1 status, MGMT promoter status, radiation therapy and 1p/19q codeletion) and the risk score. We found that the risk score was higher in older patients (age > 40 years), and in patients with grade III, IDH1 wild‐type, MGMT promoter unmethylated, patients receiving radiation therapy, and in those with 1p/19q non‐codeletion (*P* <.05, Figure 6A‐F).

### 3.6 Immune cell infiltration in high‐ and low‐risk groups

We assessed the differences in macrophage and monocyte infiltration between different subgroups, as defined by the risk model. Figure 6G shows that the high‐risk group had significantly higher populations of macrophages and monocytes than the low‐risk group. As for the CGGA database, the results were similar to those for the TCGA (Figure 6H). It is worth mentioning that ferroptosis may regulate the tumour immune microenvironment, which has great significance for glioma therapy.

## 4 DISCUSSION

Increasing evidence has shown that ferroptosis is essential for eradicating carcinogenic cells^23^ and that the sensitivity to ferroptosis is different in various types of cancers.^24^ Cysteine production, lipid peroxidation of polyunsaturated fatty acids (PUFAs), iron metabolism and mitochondrial function are closely related to ferroptosis. Activation of Nrf2‐Keap1 signalling up‐regulates the glutamate‐cystine antiporter system, which accelerates the progression of glioma.^25^ However, the prognostic value of ferroptosis‐related genes in LGG has yet to be clarified. In this study, we identified 12 ferroptosis‐related genes to construct a prognostic risk model. Furthermore, the risk score was an independent prognostic biomarker in patients with LGG, which was closely related to the clinical features and immune functions.

By focusing on the functions of the 12 genes, VDAC2, MAP3K5, DNAJB6 and CHMP5 were confirmed as protective genes. Erastin is the most commonly used inducer of ferroptosis. It induces mitochondrial dysfunction, release of ROS and ultimately promotes ferroptosis by directly binding to VDAC2.^7^ High expression of VDAC2 was associated with a longer OS and negatively correlated with glioma grades.^26^ DNAJB6 is an HSP40 family protein that has significant influence on the inhibition of tumour growth and metastasis. Jiang et al found that DNAJB6 plays an important role in ferroptosis, and its expression is down‐regulated in the oesophageal carcinoma tissues.^27^ In addition, the overexpression of DNAJB6 leads to radiosensitization of glioblastoma cells.^28^ Among the other eight risk‐associated genes, NFE2L2, also known as Nrf2, mediated oxidative stress and inflammatory response.^29^, ^30^ Activation of Nrf2‐Keap1 signalling up‐regulates system Xc‐, which is critical for glutathione peroxidase 4 (GPX4). GPX4 negatively regulates ferroptosis by limiting ROS production and reducing iron intake.^25^ Further, the diosgenin‐mediated degradation of NFE2L2 can prevent temozolomide (TMZ) resistance in GBM.^31^ MT3, a neuronal growth inhibitory factor (GIF), is mainly expressed in the central nervous system (CNS). It has a cytoprotective effect on glioma through the inhibition of apoptosis.^32^ HSPB1 correlated with poor outcomes and promoted the proliferation of glioma cells by facilitating an anti‐oxidative response.^33^ Previous studies have shown that HSPB1 is a negative regulator of the erastin‐mediated ferroptosis.^34^ ARNTL/BMAL1 is associated with molecular circadian rhythms. A recent study demonstrated that the inhibition of the ARNTL‐EGLN1‐HIF1A pathway can facilitate ferroptosis.^35^ We found that most genes of the prognostic signature played an important role in ferroptosis, which is consistent with previous studies.

System Xc‐ mediates the occurrence of ferroptosis by affecting glutamate uptake and glutathione synthesis.^7^, ^36^ Previous studies have shown that when glutamate is deficient, glutamate synthesis is blocked, or system Xc‐ is inhibited, the production of ROS and lipid peroxides decreases, which further reduces the incidence of ferroptosis.^37^ Consistently, the results of functional enrichment revealed that the terms of glutamate metabolic process, glutathione peroxidase activity, regulation of iron ion transmembrane transport and immune response were mainly related to the risk model. Increasing evidence suggests that ferroptosis is associated with tumour immunity.^13^, ^38^ According to known evidence, the microenvironment of glioma is different from most solid tumours, with monocytes (macrophages and microglia) as the majority of non‐neoplastic cells.^39^ Glioma secretes chemokines to recruit microglia and peripherally derived monocytes, which make up as much as 30%–50% of the tumour tissue,^40^ and are then differentiated into tumour‐associated macrophages (TAM). TAM plays important role in promoting tumour angiogenesis, suppressing anti‐tumour immune responses and assisting glioma proliferation and invasion.^41^ Interestingly, we found that macrophages and monocytes had higher fractions in the high‐risk group. However, there is little knowledge of the potential modulation between ferroptosis and immune regulation.

Age, grade, radiation therapy, IDH1 status, 1p/19q codeletion, MGMT promoter methylation and the risk score were significantly associated with OS in the univariate Cox regression analysis. Furthermore, multivariate Cox analysis showed that the risk score was an independent prognostic factor. The risk stratification of LGG is carried out according to age, surgical resection range, tumour volume, pre‐operative neurological function and IDH1 status etc.^42^, ^43^ To personalize OS prediction, we combined the risk score with clinical factors to create a nomogram in LGG patients. In recent years, there have been several prediction models for glioma.^44^, ^45^ One strength of our study was that the ferroptosis‐related genes of the prognostic model were closely related to the grade, TMA resistance and radiosensitization in LGG patients. This result suggests that the process of ferroptosis plays an important role in tumour differentiation and treatment sensitivity. The C‐index (0.817) and calibration plots showed that our nomogram had excellent predictive power. Therefore, physicians can apply the nomogram to improve the accuracy of identifying high‐risk patients and realize accurate treatment.

In conclusion, we applied TCGA and CGGA data sets to construct and verify the prognostic signature of ferroptosis‐related genes, which have the potential to predict the prognosis of LGG. In addition, we will verify the reliability of the prognostic signature in the future through in vitro and in vivo experiments. Further study on the mechanism of ferroptosis would be helpful in providing new targets for the treatment of LGG.

## CONFLICT OF INTEREST

The authors confirm that there are no conflicts of interest.

## AUTHOR CONTRIBUTIONS

**Yi Zheng:** Data curation (equal); Investigation (equal); Writing‐original draft (supporting). **Qiang Ji:** Methodology (equal); Software (equal); Validation (supporting). **Lei Xie:** Formal analysis (equal); Software (supporting); Writing‐original draft (supporting). **Can Wang:** Data curation (equal); Software (equal). **ChunNa Yu:** Investigation (equal); Validation (supporting); Visualization (equal). **YaLi Wang:** Data curation (supporting); Formal analysis (equal). **Jing Jiang:** Visualization (equal); Writing‐original draft (supporting). **Feng Chen:** Data curation (equal); Project administration (equal); Supervision (lead). **Wenbin Li:** Conceptualization (lead); Funding acquisition (lead); Project administration (equal); Writing‐original draft (equal).

## Supporting information

Table S1

## Data Availability

The data that support the findings of this study are available from the corresponding author upon reasonable request.

## References

[1] Wang TJC , Mehta MP . Low‐grade glioma radiotherapy treatment and trials. Neurosurg Clin N Am. 2019;30:111‐118. 30470398 10.1016/j.nec.2018.08.008

[2] Lapointe S , Perry A , Butowski NA . Primary brain tumours in adults. Lancet. 2018;392:432‐446. 30060998 10.1016/S0140-6736(18)30990-5

[3] Chang SM , Cahill DP , Aldape KD , et al. Treatment of adult lower‐grade glioma in the era of genomic medicine. Am Soc Clin Oncol Educ Book. 2016;35:75‐81. 27249688 10.1200/EDBK_158869

[4] Eckel‐Passow JE , Lachance DH , Molinaro AM , et al. Glioma groups based on 1p/19q, IDH, and TERT promoter mutations in tumors. N Engl J Med. 2015;372:2499‐2508. 26061753 10.1056/NEJMoa1407279 PMC4489704

[5] Reifenberger G , Wirsching H‐G , Knobbe‐Thomsen CB , et al. Advances in the molecular genetics of gliomas – implications for classification and therapy. Nat Rev Clin Oncol. 2017;14:434‐452. 28031556 10.1038/nrclinonc.2016.204

[6] Komori T , Muragaki Y , Chernov MF . Pathology and genetics of gliomas. Prog Neurol Surg. 2018;31:1‐37. 29393190 10.1159/000466835

[7] Dixon S , Lemberg K , Lamprecht M , et al. Ferroptosis: an iron‐dependent form of nonapoptotic cell death. Cell. 2012;149:1060‐1072. 22632970 10.1016/j.cell.2012.03.042 PMC3367386

[8] Lanzardo S , Conti L , Rooke R , et al. Immunotargeting of antigen xCT attenuates stem‐like cell behavior and metastatic progression in breast cancer. Cancer Res. 2016;76:62‐72. 26567138 10.1158/0008-5472.CAN-15-1208

[9] Lewerenz J , Hewett SJ , Huang Y , et al. The cystine/glutamate antiporter system x(c)(‐) in health and disease: from molecular mechanisms to novel therapeutic opportunities. Antioxid Redox Signal. 2013;18:522‐555. 22667998 10.1089/ars.2011.4391 PMC3545354

[10] Conrad M , Sato H . The oxidative stress‐inducible cystine/glutamate antiporter, system x (c) (‐): cystine supplier and beyond. Amino Acids. 2012;42:231‐246. 21409388 10.1007/s00726-011-0867-5

[11] Chen L , Li X , Liu L , et al. Erastin sensitizes glioblastoma cells to temozolomide by restraining xCT and cystathionine‐gamma‐lyase function. Oncol Rep. 2015;33:1465‐1474. 25585997 10.3892/or.2015.3712

[12] Chen D , Fan Z , Rauh M , et al. ATF4 promotes angiogenesis and neuronal cell death and confers ferroptosis in a xCT‐dependent manner. Oncogene. 2017;36:5593‐5608. 28553953 10.1038/onc.2017.146 PMC5633655

[13] Liu H‐J , Hu H‐M , Li G‐Z , et al. Ferroptosis‐related gene signature predicts glioma cell death and glioma patient progression. Front Cell Dev Biol. 2020;8:538. 32733879 10.3389/fcell.2020.00538 PMC7363771

[14] Cheng J , Fan YQ , Liu BH , et al. ACSL4 suppresses glioma cells proliferation via activating ferroptosis. Oncol Rep. 2020;43:147‐158. 31789401 10.3892/or.2019.7419 PMC6912066

[15] Ashburner M , Ball CA , Blake JA , et al. Gene ontology: tool for the unification of biology. The gene ontology consortium. Nat Genet. 2000;25:25‐29. 10802651 10.1038/75556 PMC3037419

[16] Gene OC . Gene ontology consortium: going forward. Nucleic Acids Res. 2015;43:D1049‐1056. 25428369 10.1093/nar/gku1179 PMC4383973

[17] Kanehisa M , Goto S . KEGG: Kyoto encyclopedia of genes and genomes. Nucleic Acids Res. 2000;28:27‐30. 10592173 10.1093/nar/28.1.27 PMC102409

[18] Goeman JJ . L1 penalized estimation in the Cox proportional hazards model. Biom J. 2010;52:70‐84. 19937997 10.1002/bimj.200900028

[19] Subramanian A , Kuehn H , Gould J , Tamayo P , Mesirov JP . GSEA‐P: a desktop application for Gene Set Enrichment Analysis. Bioinformatics. 2007;23:3251‐3253. 17644558 10.1093/bioinformatics/btm369

[20] Hanzelmann S , Castelo R , Guinney J . GSVA: gene set variation analysis for microarray and RNA‐seq data. BMC Bioinformatics. 2013;14:7. 23323831 10.1186/1471-2105-14-7 PMC3618321

[21] Greenland S , Daniel R , Pearce N . Outcome modelling strategies in epidemiology: traditional methods and basic alternatives. Int J Epidemiol. 2016;45:565‐575. 27097747 10.1093/ije/dyw040 PMC4864881

[22] Miao Y‐R , Zhang Q , Lei Q , et al. ImmuCellAI: a unique method for comprehensive T‐cell subsets abundance prediction and its application in cancer immunotherapy. Adv Sci. 2020;7:1902880. 10.1002/advs.201902880 PMC7141005 32274301

[23] Dixon SJ . Ferroptosis: bug or feature? Immunol Rev. 2017;277:150‐157. 28462529 10.1111/imr.12533

[24] Xu T , Ding W , Ji X , et al. Molecular mechanisms of ferroptosis and its role in cancer therapy. J Cell Mol Med. 2019;23:4900‐4912. 31232522 10.1111/jcmm.14511 PMC6653007

[25] Fan Z , Wirth A‐K , Chen D , et al. Nrf2‐Keap1 pathway promotes cell proliferation and diminishes ferroptosis. Oncogenesis. 2017;6:e371. 28805788 10.1038/oncsis.2017.65 PMC5608917

[26] Zhou K , Yao Y‐L , He Z‐C , et al. VDAC2 interacts with PFKP to regulate glucose metabolism and phenotypic reprogramming of glioma stem cells. Cell Death Dis. 2018;9:988. 30250190 10.1038/s41419-018-1015-x PMC6155247

[27] Jiang B , Zhao YongQiang , Shi MO , et al. DNAJB6 promotes ferroptosis in esophageal squamous cell carcinoma. Dig Dis Sci. 2020;65:1999‐2008. 31701262 10.1007/s10620-019-05929-4 PMC7297805

[28] Meshalkina DA , Shevtsov MA , Dobrodumov AV , et al. Knock‐down of Hdj2/DNAJA1 co‐chaperone results in an unexpected burst of tumorigenicity of C6 glioblastoma cells. Oncotarget. 2016;7:22050‐22063. 26959111 10.18632/oncotarget.7872 PMC5008343

[29] Kontostathi G , Zoidakis J , Makridakis M , et al. Cervical cancer cell line secretome highlights the roles of transforming growth factor‐beta‐induced protein ig‐h3, peroxiredoxin‐2, and NRF2 on cervical carcinogenesis. Biomed Res Int. 2017;2017:4180703. 28261610 10.1155/2017/4180703 PMC5316418

[30] Ju Q , Li X , Zhang H , et al. NFE2L2 is a potential prognostic biomarker and is correlated with immune infiltration in brain lower grade glioma: a pan‐cancer analysis. Oxid Med Cell Longev. 2020;2020:3580719. 33101586 10.1155/2020/3580719 PMC7569466

[31] Rajesh Y , Biswas A , Kumar U , et al. Targeting NFE2L2, a transcription factor upstream of MMP‐2: a potential therapeutic strategy for temozolomide resistant glioblastoma. Biochem Pharmacol. 2019;164:1‐16. 30885764 10.1016/j.bcp.2019.03.025

[32] Cho YH , Lee S‐H , Lee S‐J , et al. A role of metallothionein‐3 in radiation‐induced autophagy in glioma cells. Sci Rep. 2020;10:2015. 32029749 10.1038/s41598-020-58237-7 PMC7005189

[33] Ye H , Huang H , Cao F , et al. HSPB1 enhances SIRT2‐mediated G6PD activation and promotes glioma cell proliferation. PLoS One. 2016;11:e0164285. 27711253 10.1371/journal.pone.0164285 PMC5053603

[34] Sun X , Ou Z , Xie M , et al. HSPB1 as a novel regulator of ferroptotic cancer cell death. Oncogene. 2015;34:5617‐5625. 25728673 10.1038/onc.2015.32 PMC4640181

[35] Yang M , Chen P , Liu J , et al. Clockophagy is a novel selective autophagy process favoring ferroptosis. Sci Adv. 2019;5:eaaw2238. 31355331 10.1126/sciadv.aaw2238 PMC6656546

[36] Yang W , SriRamaratnam R , Welsch M , et al. Regulation of ferroptotic cancer cell death by GPX4. Cell. 2014;156:317‐331. 24439385 10.1016/j.cell.2013.12.010 PMC4076414

[37] Galluzzi L , Bravo‐San Pedro JM , Vitale I , et al. Essential versus accessory aspects of cell death: recommendations of the NCCD 2015. Cell Death Differ. 2015;22:58‐73. 25236395 10.1038/cdd.2014.137 PMC4262782

[38] Stockwell BR , Jiang X . A physiological function for ferroptosis in tumor suppression by the immune system. Cell Metab. 2019;30:14‐15. 31269423 10.1016/j.cmet.2019.06.012 PMC6944065

[39] Hambardzumyan D , Gutmann DH , Kettenmann H . The role of microglia and macrophages in glioma maintenance and progression. Nat Neurosci. 2016;19:20‐27. 26713745 10.1038/nn.4185 PMC4876023

[40] Morantz RA , Wood GW , Foster M , et al. Macrophages in experimental and human brain tumors. Part 2: studies of the macrophage content of human brain tumors. J Neurosurg. 1979;50:305‐311. 422981 10.3171/jns.1979.50.3.0305

[41] Domingues P , González‐Tablas M , Otero Á , et al. Tumor infiltrating immune cells in gliomas and meningiomas. Brain Behav Immun. 2016;53:1‐15. 26216710 10.1016/j.bbi.2015.07.019

[42] Nabors LB , Portnow J , Ammirati M , et al. NCCN guidelines insights: central nervous system cancers, version 1.2017. J Natl Compr Canc Netw. 2017;15:1331‐1345. 29118226 10.6004/jnccn.2017.0166

[43] Daniels TB , Brown PD , Felten SJ , et al. Validation of EORTC prognostic factors for adults with low‐grade glioma: a report using intergroup 86‐72‐51. Int J Radiat Oncol Biol Phys. 2011;81:218‐224. 21549518 10.1016/j.ijrobp.2010.05.003 PMC3151343

[44] Qin Z , Zhang X , Chen Z , Liu N . Establishment and validation of an immune‐based prognostic score model in glioblastoma. Int Immunopharmacol. 2020;85:106636. 32534425 10.1016/j.intimp.2020.106636

[45] Tu Z , Wu L , Wang P , et al. N6‐methylandenosine‐related lncRNAs are potential biomarkers for predicting the overall survival of lower‐grade glioma patients. Front Cell Dev Biol. 2020;8:642. 32793593 10.3389/fcell.2020.00642 PMC7390977

